# Reducing the equity gap in child health care and health system reforms in Latin America

**DOI:** 10.1186/s12939-021-01617-w

**Published:** 2022-02-23

**Authors:** Daniel Maceira, Luisa Brumana, Joaquín González Aleman

**Affiliations:** 1 University of Buenos Aires, Economics Department, Independent Researcher National Council for Scientific and Technical Research (CONICET) and Senior Researcher Center for the Study of State and Society (CEDES), Buenos Aires, Argentina; 2 Regional Health Advisor, UNICEF Regional Office for Latin America and the Caribbean (2013-2018), Panama, Republic of Panama; 3 Regional Social Policy Advisor, UNICEF Regional Office for Latin America and the Caribbean (2013-2017), Panama, Republic of Panama

**Keywords:** Health care outputs, Social determinants, Equity gaps, Latin America

## Abstract

**Background:**

During the first decade of the current century, Latin American countries have shown high and consistent economic growth rates, increasing per capita GDP and reducing poverty. Social indicators improved in even the poorest and least equitable countries in the region. In terms of health care results, marked advances were made in infant mortality rates.

**Objective:**

The aim of this paper is to identify if decreasing poverty rates in Latin America and the Caribbean during the first decade of the century have had an effect on health inequality, specifically by reducing the health care equity gap and, if so, whether that trend and its effects were distributed evenly at the sub-national level.

**Methods:**

Basic statistical tools were applied to national and sub-national administrative data for eleven Latin American countries (Argentina, Belize, Bolivia, Brazil, Colombia, Dominican Republic, El Salvador, Mexico, Nicaragua, Peru, and Uruguay) to compare the evolution of a set of social determinants with a classic health care outcome, such infant mortality) during the period 1995–2012. This document proposes a set of indicators to analyze relative evolution of results and convergence to equity, and to discuss general trends in health care reforms across the region.

**Results:**

The document shows a correspondence between poverty reduction, and improvement of health care indicators at a regional level, though national differences persist. In some cases, like Brazil and Peru, the reduction in infant mortality rates is coupled with significant movements towards health equity. This trend is different in Bolivia, where the drop in poverty is not followed by better outcomes in poor departments. At the same, results are not necessarily linked to health systems organization and/or specific reforms. For instance, both Brazil and Peru pursue in applying decentralized solutions, although the incentive mechanisms are quite different: the former has a supply side structure at the public provision level while the latter has implemented mixed payment systems.

**Conclusion:**

While some of the same instruments and measures of effectiveness in health care reforms appear across the region, specific impact evaluations should be performed. To reduce the equity gap in Latin America requires not only major improvements in social determinants but also the design and implementation of sound institutional policy and more robust regulatory frameworks (institutional determinants) so that more resources yield better practices.

## Introduction

Between 1995 and 2015, per capita income doubled in the Latin America and Caribbean region; infant mortality and under-five mortality rates dropped by more than half (54 and 55%, respectively). The United Nations Millennium Declaration has served as a stimulus to address the needs of the underprivileged, among them unresolved issues related to the health system.^1^

Nevertheless, the differences between and within countries are extremely wide. Though progress has been significant, an agenda linked to the Sustainable Development Goals (SDG) capable of increasing equity in the future is required.

In this framework, debate on universal health coverage takes on new importance, as does establishing spaces for an informed debate on how to define and measure the right to access to proper health services in terms of issues like population covered and package of services and interventions guaranteed. Crucial as well is the structure of financial protection schemes to guarantee those rights in the long term.

In this context, children emerge as a priority group, and a number of regional initiatives are geared to guaranteeing them the best health care coverage possible. Most such initiatives attempt to strengthen pre-existing institutional instruments, though others entail new programs for children and their specific needs. All these initiatives propose innovations in terms of financing, insurance, and program management.

The history of attempts in Latin America and the Caribbean to address the population’s health is long and complex, yet there is no distinct regional model of institutional organization. Over the years several initiatives have been implemented, some of them overlapping within a segmented health care system. Public provision of services, Social Insurance institutions and private sector participation (as insurers or providers of services funded by out-of-pocket resources) focus on different social groups, levels of coverage and benefits, and access and quality of care vary with income group and demographic characteristics.^2^

This article brings an equity perspective to an analysis of trends in health indicators in a group of Latin American countries. The goal is to assess whether countries that reduced their infant mortality rates also reduced their health equity gap. If so, we identify health policies or reform characteristics that made a positive contribution to improvements in health equity.

The next section provides an overview of the pertinent literature as a framework for our research and identifies the methodological challenges we faced. In section three, a working methodology is proposed; its results are presented in section four. Section five offers a discussion to align policies and results.

## Background and research questions

Performing a comparative analysis of the evolution of health outcomes over time at the sub-national level in Latin America is a challenging task. In a number of countries, health outcomes are not included in the information requested in household surveys, which means that it is not possible to compare administrative data on health performance at sub-national levels to data on income group. Our review of the literature, then, was geared to identifying alternative approaches to guide our equity analysis.

Barros et al. [1] proposes an indicator to measure inequality in access to health care by gender, wealth, education level, and ethnicity. The methodology rests on the weighted Filmer and Pritchett index applied to household surveys, and identifies household possessions (radio, TV, refrigerator, etc.) and data on living conditions as a proxy of wealth. The article compares that index to a Composite Coverage Index (CCI) of access, which includes the use of contraceptives, professionally assisted births, measles vaccine coverage, and oral rehydration therapy.

Similarly, Victora et al. [2] evaluates trends in health coverage on the basis of household surveys in thirty-five low and middle-income countries in Africa, Asia, and Latin America and the Caribbean. The goal is to monitor changes in a CCI that is based on coverage of childbirth care, measles vaccination, and insecticide treated mosquito nets, and to compare that CCI across income quintiles. The analysis shows that coverage has increased in almost every country, and that inequalities have been reduced. Furthermore, the countries with the largest gains in coverage of the poor are the ones with the most rapid economic progress during the period analyzed.

Based on household surveys in Ghana, Egypt, Brazil, and Thailand, Timaeus et al. [3] investigated to what extent differences in child mortality, morbidity, and anthropometry could be related to the environmental conditions of different socioeconomic groups. Results show that the association between socioeconomic differences and child mortality is significant in Egypt and in Brazil, but less so in Thailand and Ghana. In all countries there is a clear link between child mortality and share of the population living in urban areas. Similarly, results indicate that mortality rates amongst children residing in poor urban areas are generally similar to the rates for those living in rural areas.

Boerma et al. [4] also measures wealth on the basis of asset ownership and applies that criteria to household surveys carried out in fifty-four countries between 1990 and 2006. The article computes an aggregate coverage index on the basis of four equally-weighted areas of intervention: family planning, maternal and newborn care, immunization, and basic treatment of sick children. National CCI are compared to an ideal level of coverage, which enables measurements of health care gaps. Results in this case show that the mean gap was 43%, with an average of 54% for the poorest and 29% for the richest quintile. Child treatment gaps were higher than those for family planning and for maternal and newborn care. In most of the countries, there are large in-country differences, ranging from 20 to 30% in different Latin American countries; the gap in Central African countries is as high as 50%.

Following a different approach, Adler et al. [5] explores how contextual socioeconomic variables affect child mortality by performing an in-depth review of the literature. The text considers an array of potential determinants, such as macroeconomic variables, social environment, psychological and biological factors, as well as individual behavior. A gradient of relationships amongst different income levels was found, with higher levels of child mortality in countries with lower levels of income. Gaps are smaller in more egalitarian countries, such as the Scandinavian nations. Patterns in Western European countries as a whole are more complex.

Almeida-Filho et al. [6] conducts a biometric analysis of health inequalities in South America and the Caribbean. The research identifies several approaches to measuring inequity in relation to poverty (access to economic resources or consumption of goods), socioeconomic stratification (income, education or labor status), living conditions, gender, and ethnic origin. Unlike the previously referenced works, this one concentrates on an analysis of health results, particularly child mortality, in eleven countries during two distinct periods of time.

We will focus on the distributive changes in health outcomes during the period 1995–2012. The questions raised are: Is it possible with the existing information to find a mechanism that compares health outcomes on a sub-national level in Latin America? Is there a tendency towards increasing health equity? Is there any identifiable correlation between tendencies in equity and the characteristics of the reforms implemented?

## Methodology

Few comparable household surveys are available in Latin American countries, and the information collected does not include health outcomes. Other sources of information are the only way to obtain both income-related variables and health results reported at sub-national levels. While administrative data is less specific, its use allows the calculation of differences in poverty rates across departments/provinces/states in each country. The result of that calculation can later be associated with infant mortality rates and other indicators derived from demographic and epidemiological sources.

Based on the information available, four groups of countries were selected:

- Countries with relatively high per capita income (Argentina [7, 8], Chile, Costa Rica, Mexico [9, 10], and Uruguay [11, 12])
- Countries with per capita income at the regional average level (Brazil [13, 14], Colombia [15], Ecuador, Peru [16], the Dominican Republic [17, 18])
- Countries with relatively low per capita income (Bolivia [19], El Salvador [20, 21], Guatemala, Haiti, Honduras, Nicaragua [22])
- Countries in the English Caribbean sub-region (Belize [23, 24]).
- For each country, two time-periods with comparable information were identified during the last two decades (1995–2000 and 2007–2013).

While the mechanisms to measure infant mortality rates are largely standardized internationally, that is not the case with the metric of poverty at sub-national level. Readily comparable information is available for share of the population with unmet basic needs (Argentina, Bolivia, Colombia, Peru, and Uruguay); share of the population under the poverty line (Belize, El Salvador, Nicaragua, the Dominican Republic), and share of population living in extreme poverty (Brazil) or exposed to food poverty (Mexico). Measures of poverty are not equivalent to each other in terms of the degree of poverty or needs in each case. Nevertheless, their movements over time, when using the two selected periods and comparing them with changes in health outcomes, allow us to glimpse the association between decrease / relative increase in poverty and health outcomes, within each country, in order to assess equity between sub-national jurisdictions.

Once countries were selected and information sources identified, two health outputs were considered for analysis: infant mortality rate and adolescent pregnancy rate, as key results related to under 19 years old population groups. Country mean values, standard deviations, and maximum and minimum values within each country were identified for the first indicator, as was the percentage variation between the two time-periods available in each case. That approach allowed for cross-country comparisons, as well as learning about the existent alignment with poverty indicators.

Poverty rates at each sub-national level (province/department/state) were weighted by the number of inhabitants. The sub-national populations were then assigned to poverty quintiles, with equal population density per quintile. This exercise was performed for all the countries and for the 2 years for which information was available in order to yield two homogenous periods: 1995–2000 and 2007–2012. In those countries where more data was available, the year considered was the one most closely aligned to that of the other participant countries. Once poverty quintiles had been established, the same calculation process was applied to yield yearly infant mortality rates by poverty quintile and by year.

A health equity index was built on the basis of the set of new and comparable indicators of infant mortality rate (IMR) for each country per quintile. That index is defined as the ratio of reduction (or increment) in the infant mortality rate of the poorest quintile versus the richest, normalized to the value of ten, and takes the form:

$${\left[{{\left(\boldsymbol{IMR}\right)}_{2012\hbox{-} 1995}}^{\boldsymbol{Q1}}/{{\left(\mathbf{IMR}\right)}_{2012\hbox{-} 1995}}^{\boldsymbol{Q5}}\right]}^{\ast }10$$

Health outcomes show a reduction in the equity gap if the change in the infant mortality rate in the poorest population groups (numerator) is higher than the change in richer groups. If that is the case, the index will be higher than ten. Indexes below that value indicate widening health gaps over time.

Indexes are presented in relative terms to facilitate comparison between nations with very diverse population scales. Since poverty quintiles are calculated on the basis of population scales across sub-national units, the indicator is useful to facilitating intra-country comparisons, evolution over time, and the magnitude of the gap—and, as a result, the significance of the policy challenge.

With regards to adolescents pregnancy rates, after examining the context of each country, we have identified three main factors that affect cross-country comparisons: (a) sources of data differ across countries: in some cases, pregnancy rates arise from household surveys while in other the information belongs to administrative -hospital- records, (b) when data is originated in household sources, underestimation might become a critical issue, due to non-institutional deliveries and interruptions, (c) legal status on pregnancy interruption also differ across countries, as well as the right to achieve comprehensive sexual and reproductive education and access to traditional and modern contraceptive methods. These differences across countries heavily impact on the prevention of unwanted pregnancy, especially in adolescents. The discussion of these initiatives exceeds the goals of this article. For all these reasons, we have decided to focus our analysis on infant mortality rates.

## Results

### Infant mortality and equity gaps

For the period 1995–2012, the study identifies four groups of countries based on income where health performance improved (Table 1). Bolivia, Belize, Brazil, Peru, and Uruguay stand out within their groups, which suggests the need to further study the strategies they implemented.

**Table 1:** Evolution in Poverty and Health Indicators, 1995–2012

Indicators at initial period (1995–2000)	ARG	BEL^a^	BOL	BRA	COL	ELS	MEX	NIC	PER	RDO	URU
**Poverty population**											
National average	20.63	37.83	75.53	17.23	52.12	54.89	24.65	60.16	65.18	54.43	44.58
Average variation 1995–2012	−28.19	20.04	−35.80	−39.64	− 26.35	−24.42	−27.00	−10.51	−56.84	− 5.69	−13.42
**Under-five moratlity rate**											
National average	18.09	21.32	61.00	32.92	40.71	34.93	29.54	44.81	45.54	36.68	15.59
Standard deviation	4.57	5.30	16.01	13.16	13.95	5.17	6.09	12.46	16.85	9.84	3.98
Average variation 1995–2012	− 31.39	−42.97	−25.01	− 36.63	−19.24	−32.50	−57.93	−27.77	− 52.55	−10.07	−32.96

^a^Belize data correspond to the period 2000–2005 due to the lack of previous data

*ARG* Argentina, *BEL* Belize, *BOL* Bolivia, *BRA* Brazil, *COL* Colombia, *ELS* El Salvador, *MEX* Mexico, *NIC* Nicaragua, *PER* Peru, *RDO* Dominican Republic, *URU* Uruguay

*n.a* Not applicable

Source: Prepared by the authors

In some cases, like Brazil and Peru, the reduction in infant mortality rate is coupled with significant movement towards health equity. In both cases, the improvement in health equity is more significant in poorer states and departments. Brazil and Peru are followed by Mexico, Nicaragua, Belize, Argentina, and Uruguay. In Brazil in particular, but in Peru and Argentina as well, there is an association between decreasing infant mortality rates and health gap reductions.

The tendency in Bolivia and Belize is different. The drop in poverty in some departments in Bolivia is not matched by a parallel drop in infant mortality (though there was some improvement over time). Belize is the only country for which the data indicates an increase in poverty in some sub-national units (presumably *outliers* within very small populations) and improvement in health indicators.

The statistics establish a uniform pattern across countries: level of wealth continues to be relevant to evaluating health indicators.

**Fig. 1:**
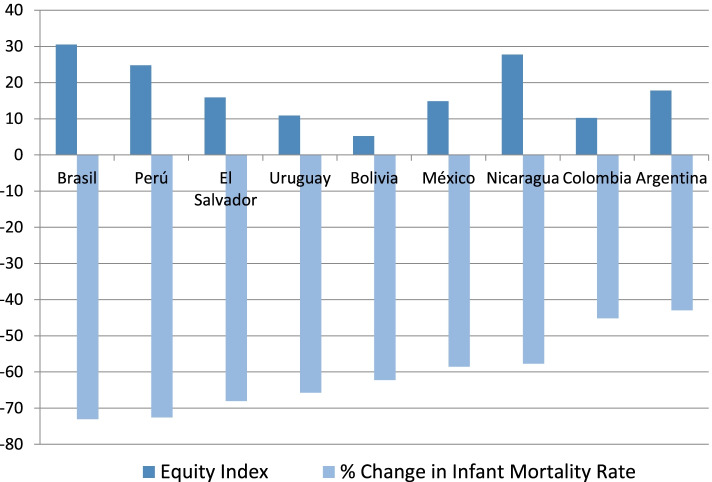
Infant Mortality Rate (% change) and Equity Index. Source: Prepared by the authors

Figure 1 presents a comparison of the two key health indicators analyzed in the study. The reduction in infant mortality rates between 1995 and 2012 in nine of the countries studied is indicated below the horizontal axis.^3^ This indicator determines the layout of the Figure, with Brazil and Peru with a drop of circa 73%. The display closes with the case of Argentina, with a reduction of approximately 43%.

In all cases except for Bolivia, equity tends to increase as infant mortality drops, and that drop is more dramatic in the poorest quintile. In the case of Colombia, the drop in infant mortality is largely uniform across poverty quintiles, though improvement is slightly greater for the poorest population. Brazil, at the other extreme, shows an indicator of equity in infant mortality of 30.5. That means that every improvement for the richest 10 % of the population is three times higher for the poorest. That gap is the largest in the region for the period 1995–2012—and it matches the absolute drop in infant mortality rate for the entire Latin America and Caribbean region.

Peru follows Brazil in magnitude of the drop in infant mortality rate with a health equity indicator of 24.8. While the drop in infant mortality is not as dramatic in Nicaragua, the equity gap there has shrunk, with greater convergence between equity and infant mortality rate at the departmental than at the national level when compared to the rest of the countries in the region.

Argentina and Mexico also reflect a major decrease in the equity gap compared to other nations such as Uruguay and Bolivia, where the drop in the infant mortality rate is larger. This implies that for the studied period, the impact of changes in the infant mortality rate was greater in the poorest sub-national spaces than in the wealthiest ones.

To summarize, improvement in health indicators (specifically infant mortality rate) does not necessarily mean a benefit for the poorest groups.

### Poverty and intra-country heterogeneity

In the case of infant mortality, there is standardized and comparable information for the different countries, including national averages and deviations, as well as minimum and maximum values at the sub-national level. The mean value reflects the situation in the country as a whole, while the deviation indicator reflects the sometimes extreme degree of disparity within each country.

Gaps compared to national averages are still quite wide in Bolivia (21.97) and Colombia (13.32), as well as in Nicaragua and the Dominican Republic (9.4 and 8.27, respectively). In all four countries, the average deviation within the country surpasses the mean intra-country deviation in the region as a whole, which is 7.21. At the other extreme, the deviations in Mexico (1.75), Argentina (2.6), and Uruguay (2.73) are below the regional domestic average.

The characteristics of each country (per capita GDP and poverty levels, access to education and urban density, among others) can explain partially these results. The international context favored elevated economic growth rates in Latin America during the first decade of the century as well. This context enabled the development of social protection policies geared to guaranteeing basic household incomes through conditional transfers, policies that had, potentially, redistributive effects. Together, the macroeconomics conditions and the social programs were conducive to better sectorial results and greater equity.

Nevertheless, our analysis does not imply a deterministic association between socioeconomic context variables and health results and their distributive effects. It suggests, rather, that two facets should be further considered and pursued: (a) the design and implementation of specific health policies, including sound mechanisms for accountability, and (b) on the basis of intra-country deviations, the impact of different managerial skills in the health care sector, whether due to the fragmented structure of health models or to the possible adverse effects of decentralization/de-concentration mechanisms.

## Discussion and policies debate

As pointed out in previous articles (Maceira [25]; Maceira [26]) and in official documents issued by international organizations and health sector researchers in the Americas, the region’s health care systems are by no means homogeneous. Segmentation into subsectors (public, social security, and private) is one pervasive feature. Formal workers contribute to social security institutions that provide care to those workers and their families, while private insurance companies cover the rich. Public systems that, despite more demanding epidemiological needs, have much lower budgets, provide health services to the poor.

These differences in financial strength, capital level, and investment in infrastructure yield differences in access and quality. Indeed, the inequalities in the health sector expose the inequalities in the region in general, and not only between countries but also within them. There is a 25-fold difference in per capita Gross Domestic Product between the two countries at the opposite ends of the region’s spectrum, the Bahamas and Haiti, and that difference has grown substantially: 20 years ago, the ratio was ten to one. These gaps translate into a difference of over 28:1 in health expenditures per capita between those two countries (World Development Indicators).

Almeida [27] sustains that during the last 20 years Latin American health care systems have revisited their organizational structures to overcome sectoral inequalities—but that remains a distant goal.

While Latin America has proven fertile ground for the implementation of health reform, there is little documentation on its impact and results. The application of social programs (conditional cash transfers, nutritional subsidies, community engagement, and others) has shed light on the needs to be addressed. Some policies act on the same social groups and in a similar direction; they run parallel to other policies focused on the financing, management, and provision of health services.

Nevertheless, similar instruments have proven successful at meeting health needs in some countries (Brazil, Peru, El Salvador, and Uruguay), regardless of differences in social determinants. A regional overlook shows both common elements and particularities in the health care reforms and programs that have been implemented, opening up debate on the effectiveness of equity-oriented interventions.

Primarily, the countries that show satisfactory health results eschew the fragmented structure characteristic of the region’s health systems in general; the Ministry of Health together with specific executive bodies (social insurance schemes or funds) pursue unified or coordinated health system organization, supported by a normative framework approved by the national legislative power. A correlate to that ideal of convergence is that health system structures attempt to guarantee common rights to all individuals.

In several countries, the pursuit of convergence is explicit. This is the case of Belize and Uruguay, two countries with relatively small populations. In the first, the strategy consists of movement towards a formal coverage scheme, an outgrowth of a traditional system based on subsidized supply, and managed by the National Health Insurance (NHI) in coordination with the Ministry of Health. Uruguay, on the other hand, reoriented a complex set of existent private health institutions (*mutuales*) through explicit health care goals and capitated payments designed by a unified National Health Fund (FONASA), under the direction of a public institution, the *Junta Nacional de Salud*. That approach was inspired by the design of the Colombian reform of the nineties, though there are some important managerial differences in terms of the instruments of control implemented, as well as the socioeconomic context (equity gaps are lower in Uruguay).

In other cases, the focus of change is solely the public sector, where wider and more effective coverage for the poor is pursued. The mid-term goal is to develop specific programs that will result in a reduction in access gaps. The Peruvian model and, to some extent, the public insurance programs in Argentina (*Programa Sumar*) and in Mexico (*Seguro Popular*) constitute a move in that direction.

Significantly, segmentation remains in the second group of countries but not in the first, that despite the intention in all cases to evolve towards more cohesive structures. Specific incentives associated with performance, explicit tracers of coverage and of “*nominalidad*” (formal enrollment) for users of public services are frequent instruments. These strategies replicate social insurances schemes—which, in some countries, have been broadly extended—within the public sphere and, hence, almost exclusively financed by the government.

The starting point in each case differs markedly in terms of resources available, capacity and quality of the public system prior to the reform, and the structure of users’ needs. All the countries are beset by monitoring structures and evaluation mechanisms that are weak at best—which constitutes a real challenge when it comes to measuring the real impact of a reform.

Brazil has sustained over the years a supply-subsidized model (*Sistema Unico de Saude, SUS*). Its achievements are largely due to coordination between decentralized levels of public management, as well as private providers, in efforts to reach the marginalized population. Inclusive social policies since the beginning of this century have served to further foster sectoral actions. Indeed, Brazil is the greatest regional example of success at decreasing the infant mortality rate and equity gap.

The health programs implemented in Bolivia and Nicaragua reflect the particular characteristics of those countries, specifically low levels of economic development. In both cases, the weight of communicable diseases is substantially higher than the regional average, and there are limited public funds to address health coverage. In response, specific programs for priority groups (maternal and child coverage schemes, indigenous population) were implemented as the main strategic goals in strengthening public health. Notwithstanding, there are some similarities with other national models, specifically regarding segmentation strength.

Several common factors surface in an analysis of the set of policy instruments deployed across health reforms: management decentralization, “nominalization” of users, explicit definition of service interventions, identification of priority groups, and performance-based payment (these instruments are summarized in Table 2).

**Table 2:** Characteristics of Health Programs

Country	Program	Specific Target^a^	Explicit package of services		Management^b^		Payment Mechanism^c^	
			Childhood	Adolesc.	Childhood	Adolesc.	Childhood	Adolesc.
**Argentina**	**Plan Nacer/ Sumar** Maternal and Child Program, later extended to the entire population without formal health insurance	**S**	**yes**	**yes**	**D**	**D**	**FB + PB**	**FB + PB**
**Uruguay**	**Seguro Nacional de Salud (SNIS) - Metas asistenciales** *National Health Insurance - Health Outcome Goals*	**G**	**yes**	**yes**	**D**	**D**	**CP**	**CP**
**Brazil**	**Plan Nacional Primera Infancia + Red Cigüeña** *National Early Childhood Program + Network Ciguena*	**S**	**no**	**no**	**D**	**D**	**FB**	**FB**
**Mexico**	**Seguro Popular/ Seguro Médico Nueva Generación** *Popular Insurance / New Generation Medical Insurance*	**S**	**yes**	**no**	**D**	**D**	**FB + PB**	**FB**
**Colombia**	**Atención integrada de enf. Prevalentes en Infancia** Comprehensive care of prevalent infant diseases	**S**	**yes**	**no**	**D**	**D**	**FB**	**FB**
**Peru**	**Programa estratégico Materno-Infantil** *Maternal-Child Strategic Program*	**S**	**yes**	**yes**	**D**	**D**	**FB + CP**	**FB + CP**
**Dominican Republic**	**Unidades de Atención Primaria (UNAP)** *Primary Care Units (PCU)*	**S**	**no**	**yes**	**C**	**D**	**FB**	**FB**
**El Salvador**	**Protección Integral de Niñez y Adolescencia** Comprehensive *Protection of Children & Adolesc.*	**S**	**no**	**no**	**C**	**C**	**FB**	**FB**
**Belize**	Seguro Nacional de Salud ***National Health Insurance***	**G**	**yes**	**no**	**C**		**CP**	
**Nicaragua**	**Programa de Atención Integral de la Niñez (PAIC)** *Comprehensive Childhood Health Care Program*	**S**	**no**	**no**	**C**	**D**	**FB**	**FB**
**Bolivia**	**Seguro Nacional Materno Infantil (SUMI)** *Mother and Child National Health Insurance*	**S**	**yes**	**no**	**D**	**n.a.**	**PB**	**FB**

^a^*S* Specific Childhood Targeted / *G* General

^b^*C* Centralized / *D* Decentralized

^c^*CP* Capitated payments / *PB* Performance-based payment / *FB* Fixed-budget

Source: Maceira [26]

Specific programs for maternal and child care are found in nine of the eleven health models studied; explicit packages of interventions for children, though not necessarily for adolescents, are important in seven cases (some of the models are based mainly on supply subsidies, while other have mixed organizations). In six cases, patient enrollment in the public subsystem and the creation of social or public insurance according to a per capita or performance-based payment model was implemented, in an approach that differs from the traditional fixed-budget model applied to public hospitals and health care centers.

Although efforts at nominalization with defined packages may be linked to improvement in coverage (Uruguay, Bolivia) and to reduction in the equity gap (Nicaragua, Argentina, and Mexico, to some extent), they cannot be isolated from other factors. More specifically, the content of guaranteed service packages is intended to reduce the financial risk facing households while also meeting their specific epidemiological need. Nevertheless, insufficient or disarticulated guarantees do not contribute to higher efficiency in the allocation of resources and have little impact on equity.

Finally, though the decentralization of health care systems is sometimes seen as an ideal way to improve sectoral resource investment and accountability, it relies greatly on enhanced management capacities in terms of priority setting, efficient and transparent resource allocation mechanisms and human resources capacity building. Failures to provide sound improvements at the local level may attempt against the efficacy of the policy.

Tracing the link between health care reforms and/or program implementations, on the one hand, and health care outcomes, on the other, constitutes a challenge for health systems research, particularly in Latin America and the Caribbean. How to separate the effects of contextual changes from those specifically associated with health care interventions? The only way to do so is with proper program evaluation methodology, which in turn requires detailed information that is not always available in low- and middle-income countries.

The methodology used has strengths and limitations. Its strengths include the ability to construct viable and replicable indicators to compensate for a lack of detailed and comparable information. Moreover, the utilization of internationally standardized health outcomes as infant mortality rates facilitates cross-national comparisons. In addition, the same methodology may be used to measure alternative equity gaps in other dimensions different than poverty, such as gender, ethnics and urban/rural. However, the indicators are still aggregated at sub-national levels, which may misallocate non-poor households residing in lower quintiles departments, or poor families residing in relatively rich areas. Overall, though, it is a suitable tool for measuring performance and equity impact.

In the debate on strategies and health programs implemented in different sectoral reforms in the region, several effective strategies were identified, among them those that reduce fragmentation and guarantee rights by schemes of financial protection such as enrollment of users at public facilities, explicit definition of rights, and the implementation of monetary incentives associated with clearly defined results. Nevertheless, the same health policy can be successful in certain contexts and neutral in others; the difference depends on how well some of its key components are implemented.

Beyond that, due to the fragmentation of health care systems, contextual variables and social programs implemented at the same time, health reforms affect different populations differently, showing that there are many differences among countries, beyond the arguments considered in this study. A comprehensive agenda on the policy making process of health systems reforms also need to address their social and political determinants, including stakeholders’ bargaining power, marginalization.

In light of this multicausality, specific and reliable evaluation programs require further developed in order to identify and isolate external factors and to provide stronger evidence on the direct effect and impact of health programs and reforms.

## Conclusions

Based on the data available, health outcomes in Latin America appear to have improved significantly during the first decade of this century. This article aims to identify if changes in national-wide infant mortality rates as key health output has been associated to reduction of equity gaps in that same indicator at the subnational level, as well as searching for potential linkages between health reforms characteristics and results.

Results show that the ability to reduce the overall infant mortality index does not necessarily reduce the equity gap between income/poverty quintiles. While that association is clear in Brazil and Peru, it is not necessarily found in all of the countries analyzed.

In addition, the spirit of the article is to contextualize the specific programs and policies on childhood, within the framework of certain innovations and the more general orientation that took place in the health sector. In that sense, reduction in equity emerge under different health systems organizations and reform efforts. Supply side models as well as demand side reforms may lead to improvements in health results, leaving room for further research in the association between other social policy interventions and the coordination to health care. Further research may focus in refining the methodology applied in measuring intra-country inequities, exploring alternative indicators for cross-country comparisons and potential convergence among intra-country regions over time.

## Data Availability

The datasets used and/or analysed during the current study are available from the corresponding author on reasonable request.

## Abbreviations

CCI: Composite Coverage Index
IMR: Infant mortality rate

## Footnotes

1 Latin American fragmented health systems impose a particular challenge for the public authority at the moment of addressing unresolved issues. When discussing potential policy actions, it is relevant to make a distinction between what is possible to achieve from the public sector as financier and provider of services, versus what kind of regulatory arrangements might be required to improve health outcomes in the private sector or social insurance schemes.

2 Some past initiatives have attempted either to coordinate health programs and policies at the regional level, beyond country boundaries, or providing general frameworks to be implemented across countries and regions. An example of the former is the Punta del Este Charter of 1961, which established health plans for the next 10 years for all countries in the region, with the exception of Cuba. On the other hand, main stream health care reforms during the nineties promoted the implementation of certain policy interventions, although they not necessarily were translated into homogeneous reforms across the region.

3 The cases of Belize and the Dominican Republic have been excluded from this comparison due to issues in their population scale structure. In the case of Belize, the extremely small population at departmental level makes any mortality indicator jump to extreme level, thus losing explanatory capacity. Something similar happens in the case of the Dominican Republic: because there are so many departments, the population of each is, with the exception of the National Capital, especially small. That means that the construction of the proposed general indicator, which relies on subnational data, is high volatile at the level of quintiles analysis.
